# Biophoton Emission Induced by Heat Shock

**DOI:** 10.1371/journal.pone.0105700

**Published:** 2014-08-25

**Authors:** Katsuhiro Kobayashi, Hirotaka Okabe, Shinya Kawano, Yoshiki Hidaka, Kazuhiro Hara

**Affiliations:** 1 The Graduate School of Systems Life Sciences, Kyushu University, Fukuoka City, Fukuoka Pref, Japan; 2 Department of Applied Quantum Physics and Nuclear Engineering, Kyushu University, Fukuoka City, Fukuoka Pref, Japan; CNR, Italy

## Abstract

Ultraweak biophoton emission originates from the generation of reactive oxygen species (ROS) that are produced in mitochondria as by-products of cellular respiration. In healthy cells, the concentration of ROS is minimized by a system of biological antioxidants. However, heat shock changes the equilibrium between oxidative stress and antioxidant activity, that is, a rapid rise in temperature induces biophoton emission from ROS. Although the rate and intensity of biophoton emission was observed to increase in response to elevated temperatures, pretreatment at lower high temperatures inhibited photon emission at higher temperatures. Biophoton measurements are useful for observing and evaluating heat shock.

## Introduction

All living organisms emit very weak light, which differs from the bioluminescence produced by luciferin-luciferase systems in fireflies, photobacteria, and hydromedusae. This light is observed as a series of individual photon emissions and the resulting luminescence, as well as the phenomenon itself, is referred to as biophoton emission [1]–[10]. The energy for this luminescence is produced when an excited biological molecule drops to a lower energy state [4], [5], and the majority of the excited biological molecules are reactive oxygen species (ROS) [11]–[15]. On being reduced, singlet oxygen, which is an ROS, also shifts to a lower energy state and emits photon [16]. Superoxide anion radical, hydrogen peroxide, and hydroxyl radicals also all oxidize biological materials and emit photons in the process [17]. The production of ROS is important because even low concentrations of ROS are toxic to living cells, as they impair membrane functioning by peroxidation of membrane lipids, reduce enzyme activity through the oxidization of peptides and carbohydrates, and promote the oxidation ornamentation of nucleic acids in DNA.

Although ROS are generated at a fixed rate by oxidation-reduction reactions during normal cellular respiration, living organisms employ a variety of mechanisms to scavenge the ROS, including antioxidant enzymes, such as superoxide dismutase (SOD) and catalase, and low-molecular weight antioxidants, such as vitamins C and E. In healthy organisms, because the concentration of ROS is maintained at very low levels by these antioxidants, the luminescence intensity of the biophotons is extremely low (≤10^3^ photons s^−1^ cm^−2^, or approximately 10^−16^ W cm^−2^) [18], [19]. However, when living organisms become stressed due to variations in temperature or other environmental perturbations, the concentration of ROS increases and strong luminescence is observed [20]–[32].

This association between stress, ROS generation [33], and biophoton emission is well documented [20]–[32], and numerous researchers, including us, consider that the stress levels of living organisms can be inferred in real time by measuring biophoton emissions [34]–[44]. We previously measured biophoton emission in plants in response to a variety of environmental stresses, including salt [45], drought [46], [47], and infestation by mites [48], [49]. In those reports we observed strong luminescence under conditions of marked growth inhibition or fatal injury. Moreover, the duration of the change in intensity, the spectrum of radiation, and the spatial distribution of the luminescence changed in accordance with the kind of stress.

In this study, we examined the effect of heat shock on stress in azuki beans. When an organism is exposed to high temperatures, proteins denature and membrane structure is disrupted [51]. Furthermore, under high temperature conditions, antioxidant enzymes and other enzymes are inactivated, promoting lipid peroxidation by ROS that are not detoxified by the enzymes [51].

Organisms have developed a variety of mechanisms to prevent injury due to high temperatures, the most common of which is the production of heat shock proteins (HSP). High levels of HSPs are synthesized when living organisms are exposed to increases in temperature of 5–10°C, or to high sub-lethal temperatures for a short time [51]. HSPs are primarily involved in the refolding of denatured proteins and the prevention of unnecessary protein aggregation. When temperatures return to normal, synthesis of HSPs ceases, but HSPs remain in the cells for several hours to several days [51]. Structural analyses of HSP genes have revealed that the structure and function of these proteins and the heat shock response are highly conserved within all living organisms. Despite the considerable number of studies on the biological response to heat shock, numerous details associated with these protective mechanisms remain unresolved. Some studies about temperature responses were performed at the beginning of biophoton research [3], [4]. However, the biological systems are very complex and more careful experiments are still necessary even now to understand the relationship between biophoton and heat shock response. And we can use the recently improved detectors that have more sensitivity in infra-red light generated by reactive oxygen species produced in metabolic process.

We therefore investigated the potential application of biophoton measurements as real time indicators of the response of living organisms to heat shock. Specifically, we first examined how biophoton emission varied in response to differences in the rate of increases in temperatures.

Biophoton signals can be measured noninvasively, in real time, and without physical contact, they are well suited for acquiring metabolic information of living organisms. We expect that measurements of biophoton emissions will increasingly be used to clarify the dynamics of heat shock responses in living organisms.

In this study, we considered that the simpler sample was suitable because the fundamental response of organisms to heat shock is so highly conserved among all living organisms. Therefore, we selected azuki bean (*Vigna angularis*) roots that have a relatively straightforward physiology as they derive all of their nutrition from carbohydrates stored in the cotyledons. Moreover, since roots grew in dark, fluorescence was avoidable in measurements. This contrasts with conditions in leaves where the heat shock response is complicated by transpiration. Furthermore, becoming more complex was expected for animal.

## Material and Methods

### Sample Preparation

The sample azuki bean (*Vigna angularis*) seeds were purchased from Nakahara Seed Co. Ltd., Japan. To induce germination, about 60 seeds were laid on wet cotton and placed in the incubator (IG-47M, Yamato Science Inc., Japan) for 24 hours under relative humidity (RH) and temperature (T) conditions of 95% and 35°C, respectively. To promote growth, RH and T were decreased to 82% and 24°C after 24 hours, respectively. To prevent photosynthesis, these incubation steps were performed in the dark. Pure water (conductivity: 0.10µS) distilled and filtered by GSH200 Aquarius purifier (Advantec Co., Japan) was used for culture solution. We selected ten germinating seeds with root lengths of 5–20 mm for one measurement, and the cotton wastes attaching to roots were removed because of their strong fluorescent. In order to investigate the responses of the intact samples which were in the same growth stage and were not heated, we used another samples for each measurement. In the measurements, we placed the sample roots into an inoperculate Petri dish (diameter: 150 mm) and added pure water in the dish until the total weight of seeds and water reached 20g.

### Experimental Setup

**Figure 1:**
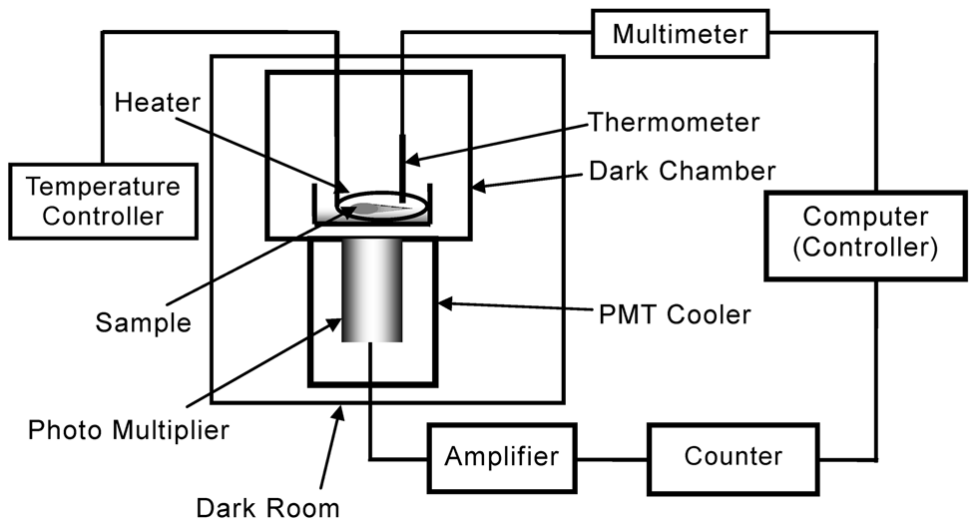
Schematic diagram of experimental setup.

Figure 1 shows a schematic view of the experimental setup. Biophotons were measured by using the photon counting system which consists of an M8784 counting board in a PC, a C3866 photon counting unit, and an R2257P photomultiplier tube cooled to −30°C in the C4877 thermoelectric cooler housing (Hamamatsu Photonics K.K., Japan). The R2257P is the low noise version of catalog model R2257. An R2257P has 46 mm^2^ photocathode and exhibits 1% or more of quantum efficiency from the wavelength of 300 nm to 900 nm and the maximum quantum efficiency is 50% in the wavelength of 600 nm. One photon counting was performed in 1 second of gate time. The dark noise at −30°C was about 190 counts per second (cps).

The sample roots in Petri dish were set above R2257P in a dark chamber. Temperature of the water in Petri dish was controlled by kp1000 (CHINO, Japan: Measurement A) and TP-4N (As one Co. Japan: Measurement B) temperature controller and its heater. The temperature near the sample roots was measured using a Pt resistance thermometer. All measurements were performed in a darkroom at ∼24°C. In addition, samples and the experimental apparatus were kept in the dark for an hour before the onset of measurements to avoid delayed luminescence (ultraweak fluorescence and phosphorescence) [31].

### Measurements

**Figure 2:**
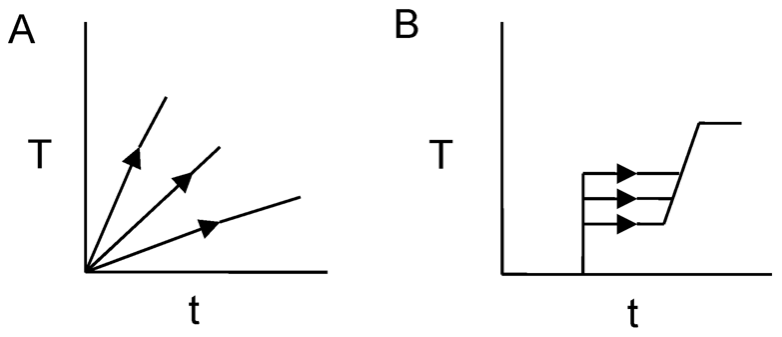
Stepped and slope-type temperature control patterns.

Sequential measurements of photon counts I (cps) and temperature T (°C) were performed under the two temperature control patterns shown in Fig. 2. Time required for one measurement for I and T was less than 2 seconds, so whole measurements are performed in almost “real time”. We were set to 40°C target temperature of these experiments. 40°C is a sufficiently high temperature to cause a heat shock, because enzyme begins to deactivation. Briefly, the changes in temperature could be described as follows:

- The sample (10 adzuki bean roots) temperature was increased from room temperature (∼25°C) to ∼40°C within from 30 minutes to 7 hours (the temperature increment rate ΔT/Δt = 0.5 ∼ 0.036 °C/min).
- Stepped and slope-type temperature control patterns. The sample (3 adzuki bean roots) temperature was increased rapidly from room temperature (∼22°C) to 25, 28, 33, or 35°C, and then maintained at that temperature for 3 hours, before being increased to ∼40°C at the same rate of increase (ΔT/Δt ∼ 0.23°C/min).

Since all sample roots grew in 24°C and were considered to adapt to 24°C as their ideal temperature for growth, we chose the start temperature of with margin of 2°.

Furthermore, in order to investigate the luminescence from the seeds that couldn’t respire, we measured it from the seeds sunk into water. The seeds can’t breathe for not exposed to air.

## Results and Discussion

**Figure 3:**
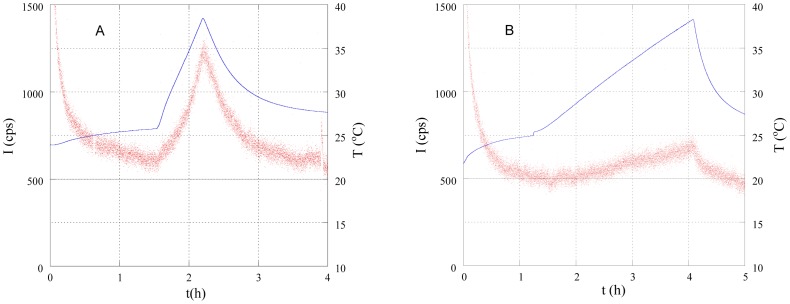
Time change of photon intensity I (dots) and temperature T (solid line) under different rates of temperature increase ΔT/Δt: (A) 0.31, (B) 0.072°C/min. The unit of photon intensity I is counts per second (cps).

**Figure 4:**
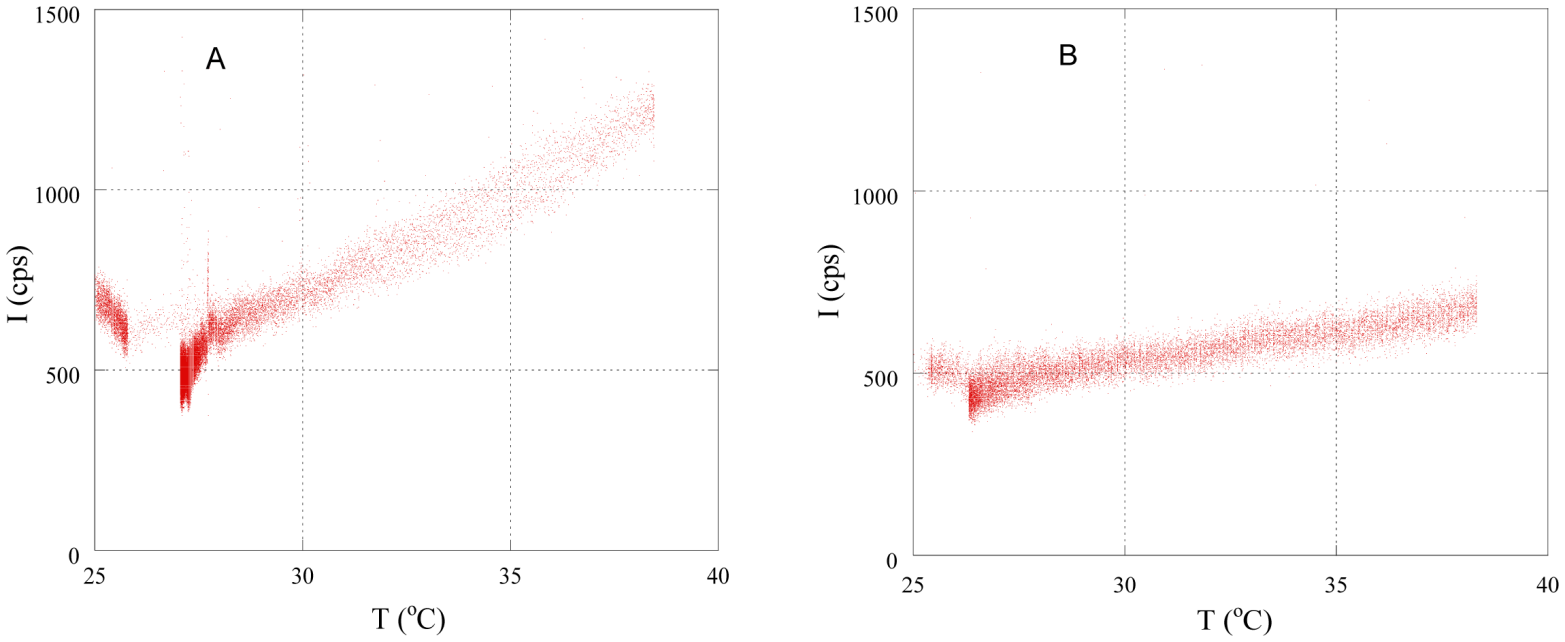
Temperature dependence of photon intensity under different rates of temperature increase ΔT/Δt: (A) 0.31, (B) 0.072 °C/min.

**Figure 5:**
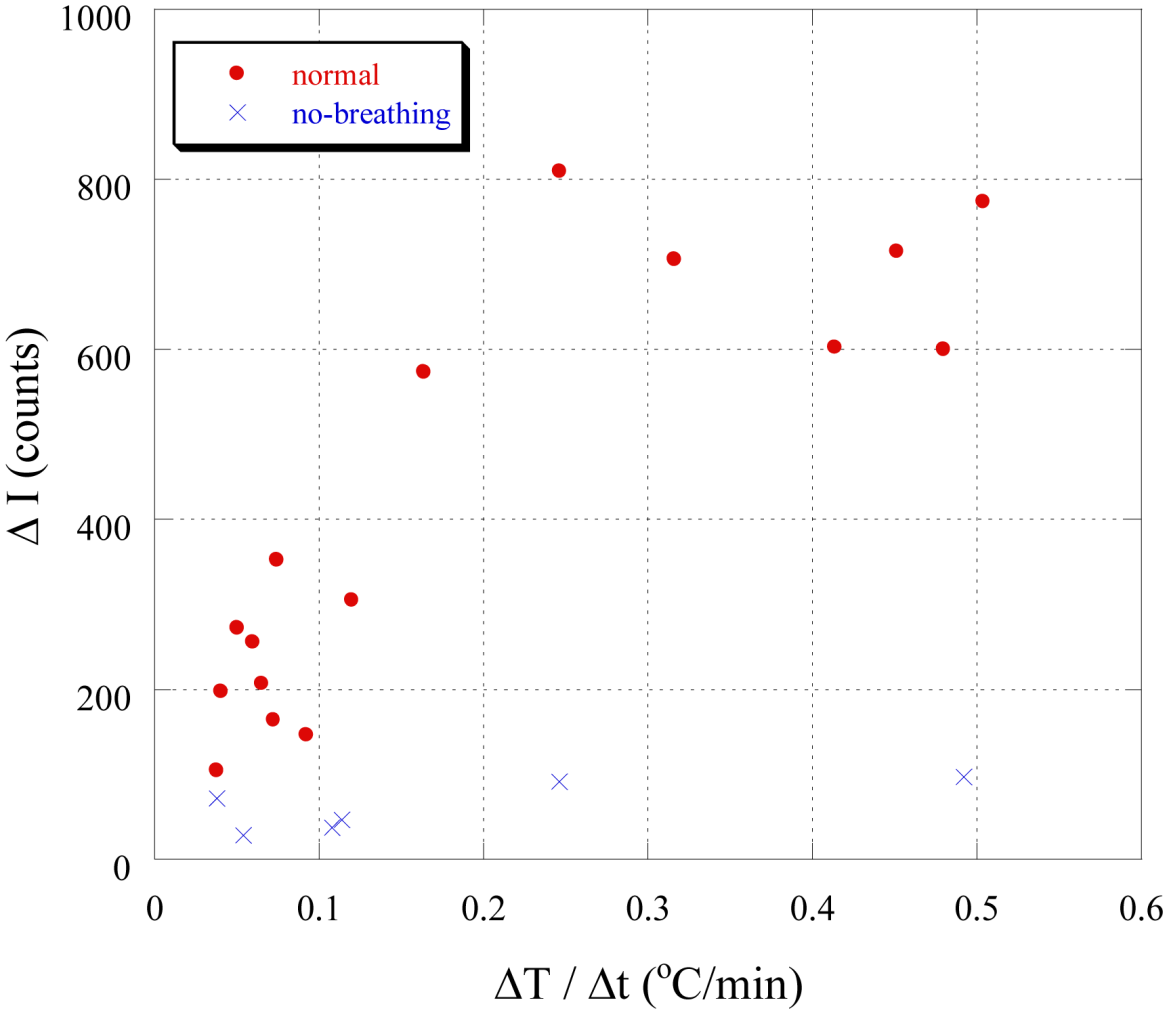
Temperature increase ΔT/Δt dependence of photon intensity changes ΔI.

Figure 3 shows the changes in the intensity of photon emissions I under the temperature control patterns shown in Fig. 2 (A). As shown in Fig. 3, the photon intensity increased during heating and decreased when heating was stopped. The intensity was also higher when the rate of the increase in temperature ΔT/Δt was large. These findings indicate that the rate of the increase in temperature ΔT/Δt is directly affects photon intensity, and this tendency is clearly shown in Fig. 4, which shows the data plotted in Fig. 3 plotted as a function of temperature. Figure 5 summarizes all of the results in Fig. 3; heating rate (°C/min) – increase of photon counts (cps). As shown in Fig. 5, in the normal samples the intensity of biophotons emitted increased with temperature, with intensities at higher rates of temperature increase ΔT/Δt being stronger than those at the same temperature, but with smaller rates of change. However, in no-breathing samples, photon counts is clearly less and constant regardless of the heating rate.

These results indicate that the stress was higher at higher temperatures and when the rate of temperature increases ΔT was higher, and that this higher stress increased ROS generation. The fact which no-breathing samples don’t emit more photons in higher temperature supports this conclusion. Previous studies on ROS generation have typically employed chemical analytical methods, and the general findings of these results are already widely known [50], [53]. However, the biophoton measurements in this study enabled real-time detection and detailed analysis of stress-induced ROS.

**Figure 6:**
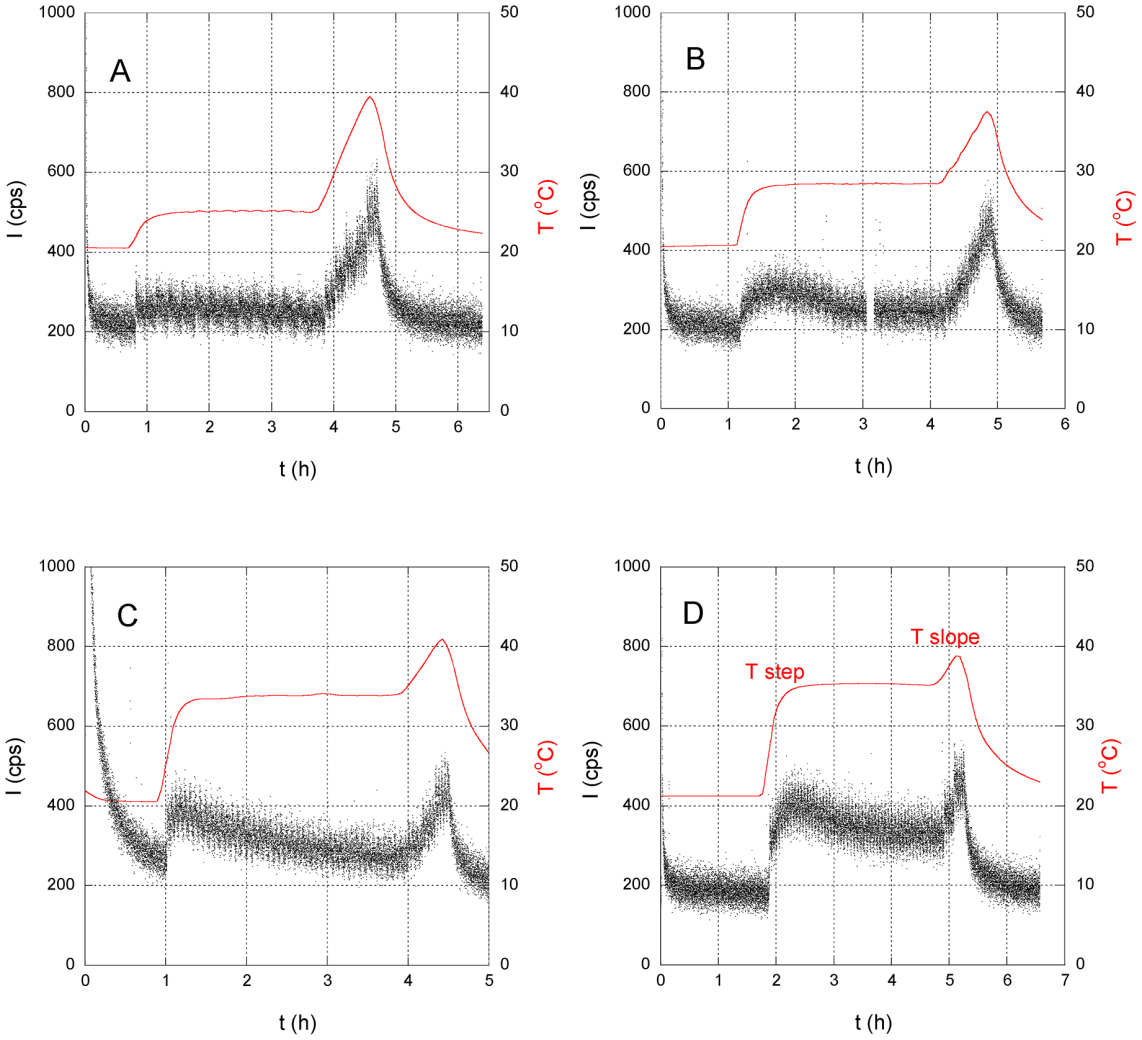
Changes in photon intensity (dots) in response to sudden changes in temperature (solid line). Temperature was increased quickly from ∼21°C to (A) 25°C, (B) 28°C, (C) 33°C or (D) 35°C, maintained for 3 h, before being increased to ∼40°C at a rate of 0.23°C/min.

To clearly demonstrate adaptation to heat shock, the beans were subjected to stepped and slope-type temperature control patterns. Figure 6 shows the changes in photon intensity in response to temperatures being increased quickly from 22°C to 25–35°C and maintained for three hours, before being increased to 40°C at a rate of 0.23°C/min. As shown in Fig. 6, photon intensity increased at each temperature step and with each increase in the rate of the temperature increase ΔT/Δt. The increase in photon intensity was proportional to the height of the temperature step (i.e., under higher heat stress). This rapid increase in ROS production has been referred to as the oxidative burst [50], and genetic studies have shown that the proteins encoded by respiratory burst oxidase homolog genes, NADPH oxidases, are the primary producers of signal transduction-associated ROS in cells. After the increase in photon intensity at the first temperature step, the photon intensity decreased gradually when temperatures remained constant. These findings implied that the increase in ROS due to heat shock initiated the adaptation process, inducing the expression of HSP, which in turn resulted in the ROS decreasing gradually over the next few hours as the adaptation process progressed.

On the other hand, for the sample that was subjected to a higher temperature step where the sample emitted many photons, the increase in photon intensity I at the temperature slope decreased as shown in Fig. 6 D. Since the rate of temperature change at the temperature slope was the same (ΔT/Δt∼0.23 °C/min) for all samples, the difference in the photon intensity originated in samples' heat resistance at that time. The heat shock following the temperature step would be associated with the production of HSPs and, consequently, with an increase in the heat resistance of the plant. Since the spectrum analysis of the photon will be effective for further investigation of the origin of the photon, we plan to improve the apparatus to measure the spectrum.

As explained above, the biophoton intensity under heat stress was always higher than it was at the reference state (before heating). In this regard, the responses of the roots were very similar to the response of roots to salt stress [45]. In that study, under conditions of low salt stress, biophoton emission intensity was lower than it was in reference plants; however, this finding was attributed to a decrease in physiological activity itself. The biophoton intensity has been shown to be dependent on the concentration of ROS, which is not only dependent on the magnitude of stress, but also on the amount of cellular respiration. Cell respiration depends on physiological activity, and in the case of salt stress it decreases as the salinity level increases [52]. Therefore, as we reported previously, photon intensity has a minimal value at certain salinity levels [45].

On the other hand, except at lethally high temperatures, cellular respiration and HSP expression both increase with temperature, implying that the intensity of biophoton emissions increases uniformly with an increase in temperature. Since respiration rate decreases under conditions of low-temperature stress, it is expected that the same decrease in respiration would be observed under conditions of low-temperature stress. In addition, the phenomenon of oxidization burst was not observed under conditions of low-temperature stress [50].

## Conclusion

Heat shock induces biophoton emission, which means that luminescence in biological tissues increases greatly when the temperature is increased rapidly. This phenomenon can be attributed to the generation of ROS, which can be used to infer the degree of stress in the organism. Biophoton measurements also facilitate noninvasive observations of stress in real time, and are thus a potentially useful method for evaluating heat shock and other stresses in living tissues.

We have improved our measurement system and can now control temperatures more precisely by microwave heating, which has enabled us to detect temperature stress responses in the order of several degrees by cyclic heating, long-term measurement and averaging. We are currently investigating the fluctuations of ROS in response to small temperature fluctuations and will report these results in the near future.
